# Quantitative analysis of the effect of tubulin isotype expression on sensitivity of cancer cell lines to a set of novel colchicine derivatives

**DOI:** 10.1186/1476-4598-9-131

**Published:** 2010-05-30

**Authors:** Chih-Yuan Tseng, Jonathan Y Mane, Philip Winter, Lorelei Johnson, Torin Huzil, Elzbieta Izbicka, Richard F Luduena, Jack A Tuszynski

**Affiliations:** 1 Department of Oncology, Cross Cancer Institute, University of Alberta, Edmonton, AB, T6G 1Z2, Canada; 2 Cancer Therapy and Research Center, The Institute for Drug Development, 14960 Omicron Drive, San Antonio, TX, 78245, USA; 3 Department of Biochemistry, University of Texas Health Science Center, San Antonio, TX, 78229, USA; 4 Department of Physics, University of Alberta, Edmonton, AB, Canada

## Abstract

**Background:**

A maximum entropy approach is proposed to predict the cytotoxic effects of a panel of colchicine derivatives in several human cancer cell lines. Data was obtained from cytotoxicity assays performed with 21 drug molecules from the same family of colchicine compounds and correlate these results with independent tubulin isoform expression measurements for several cancer cell lines. The maximum entropy method is then used in conjunction with computed relative binding energy values for each of the drug molecules against tubulin isotypes to which these compounds bind with different affinities.

**Results:**

We have found by using our analysis that *αβ*I and *αβ*III tubulin isoforms are the most important isoforms in establishing predictive response of cancer cell sensitivity to colchicine derivatives. However, since *αβ*I tubulin is widely distributed in the human body, targeting it would lead to severe adverse side effects. Consequently, we have identified tubulin isotype *αβ*III as the most important molecular target for inhibition of microtubule polymerization and hence cancer cell cytotoxicity. Tubulin isotypes *αβ*I and *αβ*II are concluded to be secondary targets.

**Conclusions:**

The benefit of being able to correlate expression levels of specific tubulin isotypes and the resultant cell death effect is that it will enable us to better understand the origin of drug resistance and hence design optimal structures for the elimination of cancer cells. The conclusion of the study described herein identifies tubulin isotype *αβ*III as a target for optimized chemotherapy drug design.

## Background

### Tubulin as a Target for Chemotherapy

Tubulin is a structural protein whose *α*/*β *hetero-dimer forms the constituent subunit of microtubules MTs [1]. MTs are critically involved in cellular processes such as mitosis, intracellular transport and cell motility. For cancer chemotherapy, tubulin is the target of some of the most successful anti-tumor drugs, such as the taxanes and the vinca alkaloids [2,3]. When the three-dimensional structure of a drug target is known [4,5], it is theoretically possible to use computational methods to design drugs that will bind specifically to that target and thereby become therapeutically useful. Since tubulin is such a successful anti-tumor drug target, and since its three-dimensional structure has been determined (including the case when it is bound to colchicines), it is logical to apply rational drug design and synthesize drugs that will target tubulin even better than presently used drugs. An important issue that has been, by and large, left unanswered is which of the several tubulin isotypes should be specifically targeted in cancer chemotherapy. The ultimate goal, therefore, is to design drugs that bind well to the over-expressed tubulin isotype and are lethal to cancer cells but not to normal cells. We have evaluated our initial approach to rational drug design based on tubulin as a target and specifically its colchicine binding site. We have chosen the colchicine site because: 1) colchicine is a drug with a long clinical history [6]; 2) the precise mechanisms of colchicine binding, including conformational effects, have been worked out better than for any other tubulin-binding drug [7-14]; 3) the synthetic chemistry of colchicine and its derivatives is simpler than that of other tubulin-binding drugs [15,16]; 4) colchicine has strong anti-mitotic activity which can be used as a standard for comparison of the derivatives that we design [17]; 5) colchicine has been used in clinical trials but, due to dose-limiting general toxicities has not been successful so far [6]; 6) tubulin isotypes differ significantly from each other in their binding to colchicine and some of its derivatives. Our hope is that by altering the structure of the drug to make it more specific for cancer cells, its therapeutic concentration can be lowered below the toxicity limit. The issue of particular importance in our study was to determine the sensitivity of cancer cells to those drugs that target one or more tubulin isoforms. As a result of this work, we have determined specific molecular targets that should both improve the efficacy and lower the general toxicity of these anti-mitotic compounds.

In the initial stage of the project we have performed computer modelling to design two series of colchicine derivatives. The first series had minor changes that were predicted to decrease the binding to tubulin while the other had side groups added in order to increase their binding affinity, in particular with respect to the isotype *αβ*III tubulin isoform that is commonly over-expressed in cancer cells [18-27] and hence was predicted to be a suitable anti-cancer target. To assist the reader in following our strategy, we will briefly discuss the issues of: (a) the colchicine binding site in tubulin and the design of colchicine derivatives, (b) cytotoxicity assays and (c) tubulin isotype expression measurements in the section of Materials and Methods. The details are discussed elsewhere [28].

### Goal

The ultimate goal of our work is to investigate the relative importance of tubulin isotypes in eliciting response of cancer cells to cytotoxic stress. Specifically, we have chosen to analyze this issue using a novel family of tubulin-binding compounds created as derivatives of colchicine. In order to understand the complex behaviour of various cancer cells exposed to these drugs, we propose to apply the maximum entropy (ME) approach [29-35] to predict the expression levels of specific isotypes of tubulin in response to cytotoxic agents introduced. Six cancer cell lines, A549, MCF7, CEM, HeLa, M006X, and M010B, are considered in this study and they were subjected to colchicine and 20 of its novel derivatives with significantly different binding affinities for each tubulin isotype, particularly *αβ*I,*αβ*II,*αβ*IIIand *αβ*IV. Tubulin is assumed to be the primary target of colchicine's action and therefore binding affinity is also assumed to correlate with the toxic effect on cells exposed to the drug, the final outcome being apoptosis. The benefit of being able to correlate expression levels of specific tubulin isotypes and the resultant cell death is that this could enable us to better understand the origin of drug resistance and hence assist us in the design of optimized structures for the elimination of cancer cells. Anti-tumor drugs that target tubulin differ in their affinities for specific tubulin isotypes. Both *αβ*II and *αβ*III are frequently over-expressed in cancer cells. Paclitaxel and vinblastine, both of which are very successful, favour the *αβ*II over the *αβ*III isotype. However, *αβ*II is very abundant in the nervous system and a few other tissues, hence side effects, such as neuropathy, commonly occur with these drugs. Tubulin isotype *αβ*III, which is much less widespread than *αβ*II, is therefore an attractive target for novel drugs. As will be demonstrated in the remainder of this paper, *αβ*III tubulin is indeed confirmed as the best molecular target within the tubulin family.

## Materials and methods

### The colchicine binding site and the design of colchicine derivatives

Unlike paclitaxel, the binding of colchicine does not result in MT stabilization; instead it results in their destabilization. Initial structures of a two-heterodimer protofilament complexed with the Stathmin-like domain of Rb3 were determined based on the information in the Protein Data Bank (PDB identifier: 1 FFX). Recently, this work was followed by an additional structure that identified colchicine as binding between the *α *and *β *tubulin molecules of the hetero-dimer itself (PDB identifier 1SA0). Ravelli *et al. *[8] identified several principal interactions between the bound colchicine and *β *tubulin. First were interactions with sheets S8 and S9, helix H7 and H8 and loop T7 (see Nogales, *et al. *[4]). During our analysis, we identified a total of 29 residues of *β *tubulin within the 6 Å cutoff from the bound colchicine. Most of them are contained in the middle of the protein. The colchicine binding site is composed of residues 235-240, 246-257, 312-316 and 347-352. Of these 29 residues, seven positions show differences among the *β *tubulin isotypes. All of the observed substitutions occur within the H7 and H8 helices, and the S8 and S9 sheets. These positions were initially identified by Ravelli *et al. *[8] as those that are displaced upon colchicine binding to the *β *tubulin. Differences here occur over a wider range of *β *isotypes, encompassing *β*III, *β*V, *β*VI, *β*VII and *β*VIII (see Table 1). Many of these substitutions are conservative, with the exception of *β*VII position Val255-Met and *β*III, *β*V and *β*VI position Ala316-Thr/Cys. Interestingly, there are four positions within the tubulin sequence alignment that have no clear consensus residue over all the *β *isotypes, one of which is position 316, which falls within the colchicine binding site and can accommodate either Val or Ile.

**Table 1:** Tubulin Isotype Interactions with Colchicine

Isotype	Substitutions at Binding Site					
*β*III	X	Cys239-Ser	X	X	Ala315-Thr	Thr351-Val
*β*V	X	Cys239-Ser	X	X	Ala315-Thr	Thr351-Val
*β*VI	Val236-Ile	Cys239-Ser	X	X	Ala315-Thr	Thr351-Val
*β*VII	X	X	Val255-Met	Val313-Ala	X	X
*β*VIII	X	X	X	Val313-Ala	X	X

When comparing the results of our modeling of the colchicine binding site to the data obtained in the 1SA0 structure, we see no obvious differences, with the exception of Cys239-Ser in *β*III, *β*V and *β*VI, which was identified by Chaudhuri, *et al. *[14] as being involved with colchicine binding through cysteine labeling studies. This position, while spatially conserved, produces an altered chemical environment, the difference being the presence of a hydroxyl or sulfhydryl moiety. A change like this could be exploited by producing covalently bound forms of a drug, under certain conditions in the form of either a disulfide or ester linkage. Additionally, the substitution Val255-Met within the *β*VII isotype might alter the positional dynamics of helix H8 and therefore influence colchicine binding in this way. Residues that are present within the interface between H7/H8 and S8/S9, prior to their displacement upon colchicine binding, may also produce interactions that impart varied stability to this region.

### Structures of the compounds

**Figure 1:**
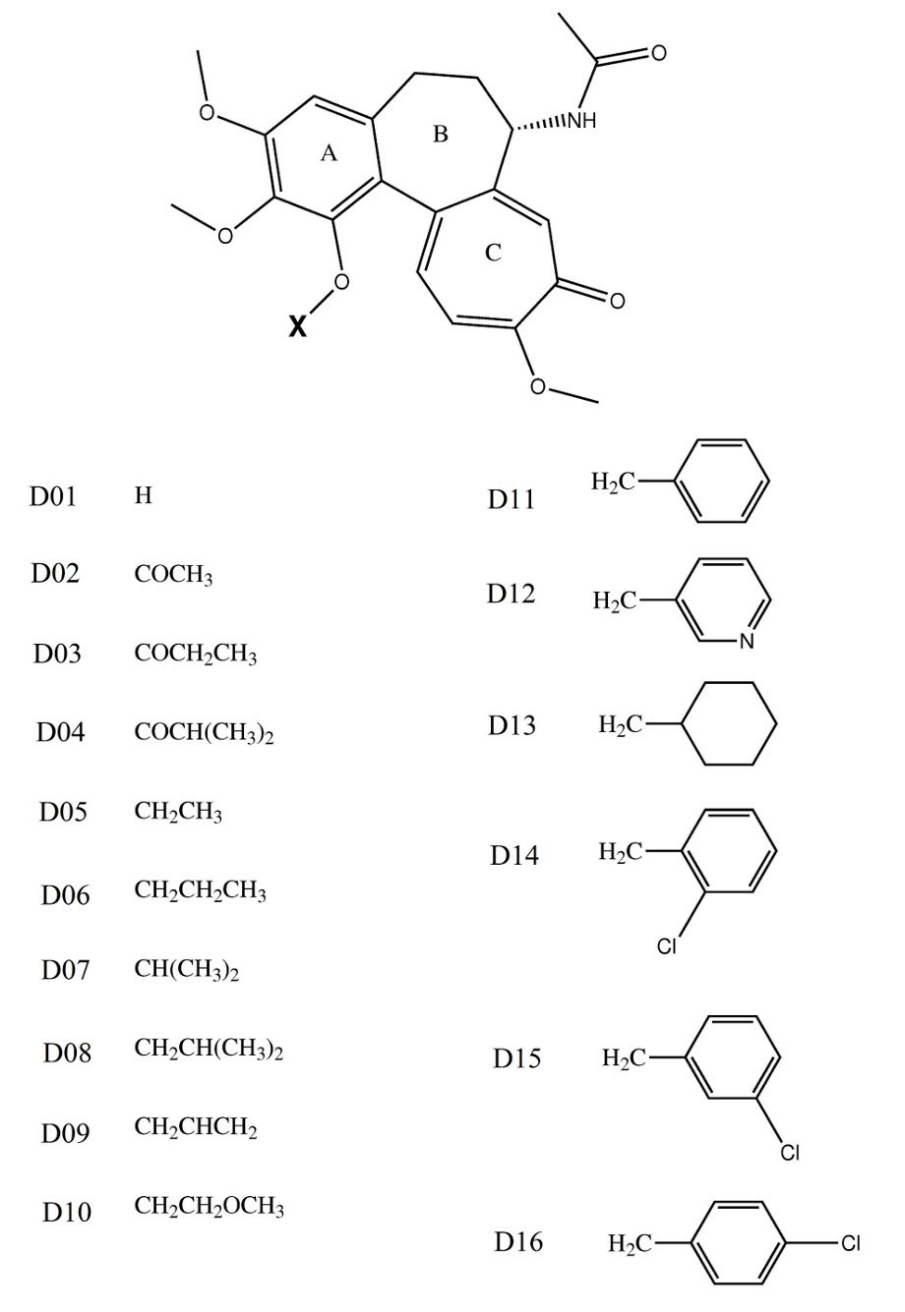
**The structures of the first class of colchicines**. The structures of the first class of colchicine analogs and the corresponding X-side groups.

**Figure 2:**
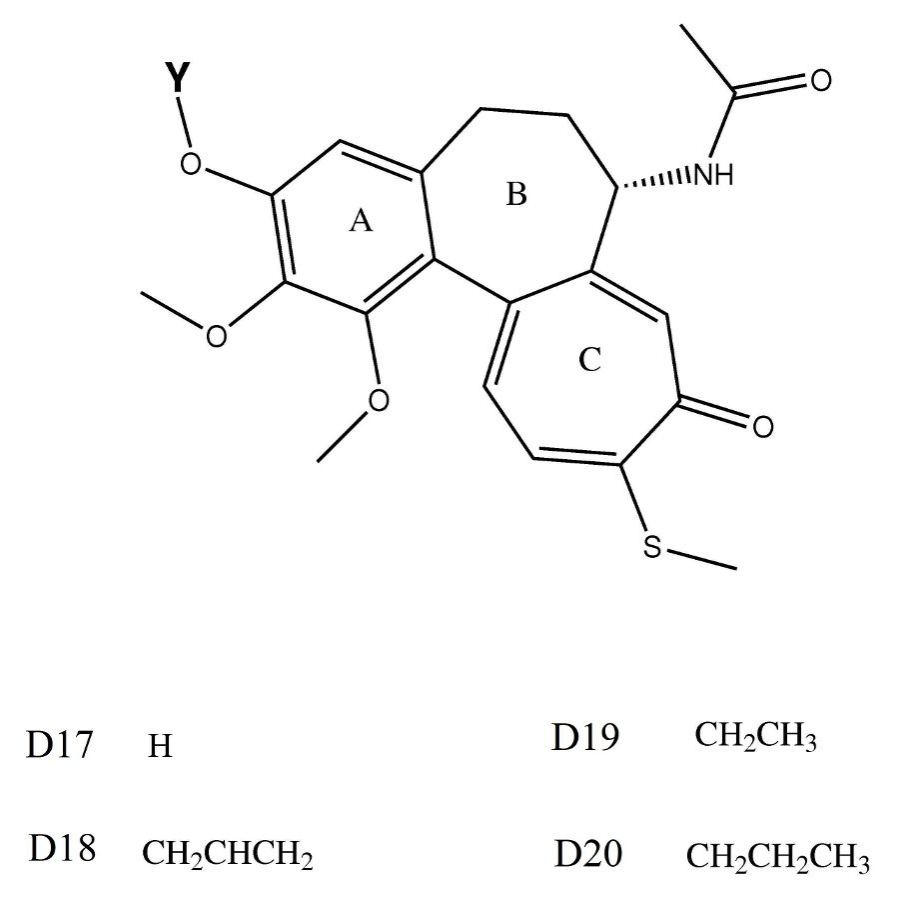
**The structures of the second class of colchicines**. The structures of the second class of colchicine analogs and the corresponding Y-side groups.

The two main classes of structural analogs of colchicine and the side groups used in this study are given in Figures 1 and 2. The substituents D1-20 were chosen based on how colchicine interacts and sits in the *β*-tubulin binding pocket. The generation and characterization of colchicine derivatives has been described previously (see [36]) based on the differences observed among the most commonly expressed human tubulin isotypes: *αβ*I, *αβ*II, *αβ*III and *αβ*IV. The colchicine derivatives were modified at either the × (C1) or Y (C3) methoxy position of the A-ring. Acylation of the common intermediates 1- or 3-demethylcolchicine afforded ester derivatives while alkylation gave ether derivatives. The general synthetic schemes for the ester and ether derivatives of colchicines were based on previously published schemes [15]. The modification to the first analog is done by replacing the -*OCH*_3 _in the *C*13 position of the *A*-ring of colchicine by different -*OX *groups. In the second analog, the -*OCH*_3 _inof colchicine are replaced by the *C*11 position of *A*-ring and the -*OCH*_3 _in the *C*-ring of colchicine are replaced by different -*OY *groups and -*SCH*_3_, respectively. All of these resulting derivative structures including colchicines that are defined as D01-D20 as appeared in figures 1 and 2 and D00 respectively are constructed using MOLDEN program [37], then optimized in the gas phase with the GAMESS-US program [38] using the Austin Model 1 (AM1) semi-empirical method [39,40] before being used in the *in silico *studies in the next section.

### *In silico *binding affinity prediction

The relative binding affinities for each of colchicine derivatives have been calculated for all major human *β *tubulin isotypes in order to determine which of them bind better to specific tubulin isotypes. The binding free energies and stabilities of colchicine analogs and derivatives, placed within the binding site of *αβ*-tubulin heterodimer solvated in a 115 A × 75 A × 75 A rectangular box of water molecules, have been evaluated using hybrid quantum mechanical/molecular mechanical (QM/MM) technique. The computer program DYNAMO [41] and GAMESS-US [38] has been used in this study. For the *α*-tubulin subunit, we used the corresponding *α*-tubulin chain 1SA0 [8] taken from the RCSB Protein Databank. Each bio-molecular system has been partitioned into two regions: (a) a small portion containing the binding site, i.e., colchicine derivative and the immediate surrounding amino acid residues; and (b) the remaining larger portion, representing the rest of the *α*/*β*-tubulin hetero-dimer. The former has been treated quantum mechanically and the latter via molecular mechanics. The reason for such a partitioning scheme is that we want to model the dynamic distribution of electrons in the binding site (through QM), and at the same time provide a more realistic model environment enclosing the binding site (through MM). In this hybrid QM/MM approach, the QM portion interacts with the MM portion and vice-versa.

**Figure 3:**
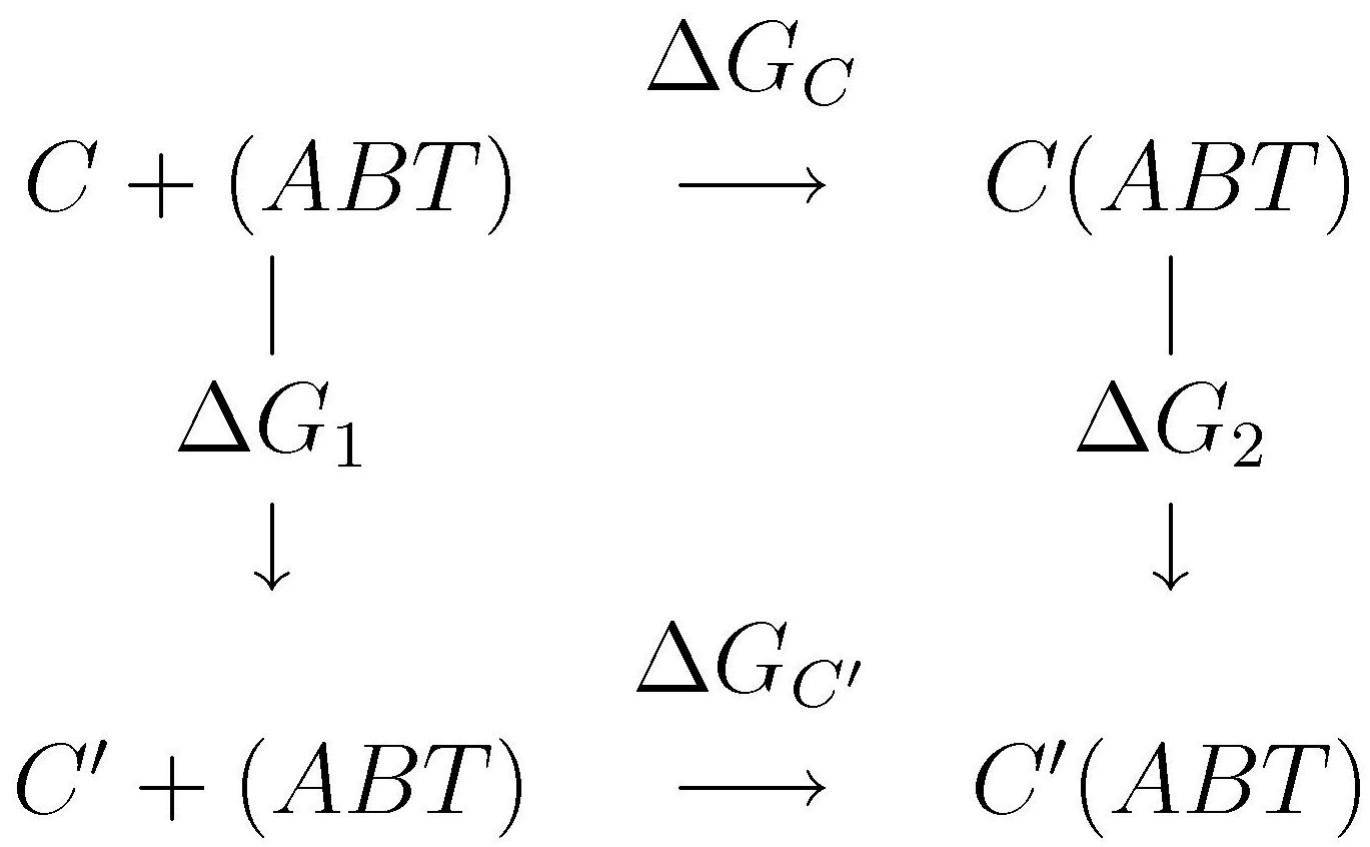
**Thermodynamic cycle approach**. The schematic plot of thermodynamic circle approach used for calculating relative binding free energy.

The thermodynamic cycle perturbation approach was proposed to overcome the difficulties related to the computational complexity of the problem [42]. Figure 3 shows the scheme that is used as the basis for the determination of the relative free energies of binding [36]. In this scheme, ABT represents the *α*-tubulin dimer, and the values of Δ*G *s represent free energy changes for the indicated processes. The relative binding of C and C' to ABT is determined by ΔΔ*G*_bind _= Δ*G*_2_-Δ*G*_1 _rather than calculating Δ*G*_*C'*_-Δ*G*_*C *_directly. Table 2 summarizes the results of the binding free energy estimates for the 20 derivatives of colchicine and colchicine itself against seven *β *tubulin derivatives. The energy values are given in kcal/mol.

**Table 2:** Relative binding free energy estimates

	I	IIa	IIb	III	IVa	IVb
**D01**	-13.2 ± 1.8	5.9 ± 1.5	-2.8 ± 1.6	1.1 ± 1.7	5.5 ± 2.6	-16.9 ± 2.7
**D02**	-9.6 ± 3.3	-24.8 ± 1.6	-7.9 ± 2.2	1.4 ± 2.2	15.1 ± 4.0	-5.4 ± 3.9
**D03**	-19.9 ± 6.2	-10.7 ± 3.9	-17.0 ± 3.9	6.6 ± 2.4	-9.5 ± 4.7	-20.7 ± 7.6
**D04**	-30.0 ± 4.8	-8.3 ± 2.1	-7.0 ± 3.9	2.2 ± 4.0	-8.2 ± 4.2	-10.0 ± 3.4
**D05**	-13.8 ± 3.4	-11.5 ± 2.0	3.5 ± 2.4	5.0 ± 1.7	-7.0 ± 3.5	-13.9 ± 2.1
**D06**	8.6 ± 4.0	4.8 ± 2.1	20.0 ± 1.7	-4.3 ± 1.3	8.1 ± 2.6	-12.7 ± 5.7
**D07**	-2.8 ± 0.8	-9.8 ± 1.7	2.6 ± 1.7	1.2 ± 1.6	1.8 ± 2.3	-10.2 ± 1.9
**D08**	-2.5 ± 2.7	-7.8 ± 1.6	1.1 ± 4.5	2.2 ± 3.0	3.5 ± 3.1	-16.5 ± 6.2
**D09**	2.9 ± 5.8	-7.0 ± 1.9	0.1 ± 3.3	0.3 ± 4.4	14.1 ± 3.7	4.7 ± 6.3
**D10**	-10.6 ± 4.6	-13.2 ± 3.0	-4.9 ± 4.3	-21.7 ± 1.8	-15.9 ± 4.1	-15.8 ± 6.1
**D11**	37.6 ± 1.6	-0.6 ± 0.8	10.7 ± 1.7	13.1 ± 0.3	3.2 ± 3.4	-19.1 ± 1.6
**D12**	-15.6 ± 1.6	-23.2 ± 2.1	-4.1 ± 1.9	-15.1 ± 1.6	-2.8 ± 2.7	-30.4 ± 3.3
**D13**	0.1 ± 2.3	-5.2 ± 3.4	9.7 ± 1.5	2.3 ± 2.2	-3.7 ± 2.3	-18.1 ± 3.6
**D14**	-0.1 ± 4.0	-11.0 ± 1.9	0.6 ± 1.5	26.3 ± 1.9	26.5 ± 2.7	-20.0 ± 6.1
**D15**	12.4 ± 3.3	-3.5 ± 1.6	-1.4 ± 0.8	6.3 ± 1.6	-4.3 ± 2.3	-4.4 ± 3.2
**D16**	-2.8 ± 4.2	-3.9 ± 0.9	5.5 ± 1.8	-9.1 ± 3.8	-9.9 ± 3.5	-15.5 ± 2.2
**D17**	7.8 ± 5.1	-1.3 ± 1.4	3.1 ± 4.3	1.9 ± 1.5	26.1 ± 3.6	-6.6 ± 3.8
**D18**	-2.1 ± 2.9	-1.1 ± 3.4	3.3 ± 2.8	16.8 ± 1.4	-5.6 ± 1.9	-10.1 ± 2.3
**D19**	-10.3 ± 3.8	3.4 ± 3.2	-11.0 ± 2.3	-0.2 ± 1.5	-15.1 ± 3.2	-6.1 ± 5.1
**D20**	-1.7 ± 5.1	0.9 ± 1.7	10.6 ± 3.5	7.8 ± 1.8	-6.4 ± 3.9	-8.4 ± 3.7

Relative binding free energy estimates of the 20 derivatives of colchicine and colchicine itself (listed in the first column) against seven tubulin isotypes listed in the top row. The energy values are given in kcal/mol and the detailed derivation is given in [36].

Computational screening gave us confidence that some of the designed derivatives are both tubulin isotype specific and that they may possess high affinity for the target making them therapeutically viable. Subsequent experimental validation using cytotoxicity assays demonstrated that some of these first generation derivatives are indeed superior to colchicine in their effects on tumor cells.

### Cytotoxicity assays

The new colchicine derivatives were tested, together with their parent compound, colchicine, on a variety of cancer cell lines in a variety of assays: cytotoxicity, induction of apoptosis, and effect on cell morphology.

For initial screening, we chose a variety of common cancer cell lines based on the diversity of their cancers of origin as well as their differing morphologies. Testing cytotoxicity of the colchicine derivatives against cells with a variety of morphologies and origins can help us determine any differences in derivative efficacy based on cell type. The cell lines we initially chose to experiment with included CEM, MCF-7, HeLa, A549, as well as M006X and M010B cells, sister glioma cell lines. Table 3 lists the cell lines used, including their origins and growth conditions.

**Table 3:** Origins and growth conditions of common cancer cell lines used for MTS cytotoxicity assays

Cell Line	Origin	Media
A549	Human lung carcinoma	RPMI with 10% fetal bovine serum (FBS)
MCF-7	Human mammary gland adenocarcinoma	RPMI with 10% FBS
CEM	Human T-lymphoblastoid from ALL	RPMI with 10% FBS
HeLa	Human cervical carcinoma	DMEM with 10% FBS
M006X	Human glioma cells	DMEM-F12 with 10% FBS and 1% Glutamine
M010B	Human glioma cells	DMEM-F12 with 10% FBS and 1% Glutamine

Derivative drug stock solutions were prepared by gravimetrically weighing out a specific amount of the compound, dissolving in DMSO, and diluting to a final volume with distilled water (final [DMSO] = 4.5%). A series of drug dilutions were then prepared to determine compound characteristics. The lab spectrophotometer was used to complete a wavelength scan of the diluted drug solution, which determined the wavelength of maximum absorbance of the drug. Five known drug concentrations were then scanned at this wavelength. These concentrations were plotted (X) versus their absorbance values at the pre-determined wavelength (Y) to get a linear graph; the slope of the line is a rough estimate of the compound's exhaustion coefficient. This coefficient can be used in conjunction with a current absorbance reading of the drug stock solution to determine the concentration of this solution whenever it is needed. Once the compound characteristics were determined, the drug was used in MTS cytotoxicity assays on our cell lines.

Cytotoxicity testing was completed using a common cell viability assay, the MTS assay. A primary MTS assay is used to test the number of cells ideal for use in the cytotoxicity assay. Cells are trypsinized, counted and set up in 7 lanes of a 96-well plate at various cell numbers (with 8 wells/replicates per lane). An eighth lane includes a media alone control. The goal of this MTS cell number test is to find the number of cells (per well in 96-well plates) that will be in exponential growth after 72 and 96 hours for adherent cell lines, and after 48 and 72 hours for suspension cell lines. Using this cell number for cytotoxicity assays allows us to find the effects of the anticancer drug on exponentially growing cells, conditions similar to those of actual tumors. The 24 hour difference in growth time between adherent and suspension cells is given to allow adherent cells time to attach to their growth surface. Cells then have time to set up and grow in a manner respective of their tumor type, allowing us to gather more accurate information of the potential responses of their tumor types to various drugs. Suspension cells, on the other hand, do not require the extra 24 hour growth period. They are plated directly in their natural tumor form (in suspension), and drugs can therefore be added to cells in this state with no set up time.

For cytotoxicity testing, adherent cells were plated into 9 lanes of a 96 well plate at the pre-determined cell number (a tenth lane includes a media alone control). Twenty-four hours later, various concentrations of drug were added to eight of the lanes containing cells (the ninth lane containing cells is a control). At this time, drug solutions were prepared with suspension cell lines at an appropriate cell concentration (as determined in the MTS cell number test), and the suspension cell line experiments were plated. All cell lines were then grown with the drug for 48 and 72 hours. Cell viability was determined using the CellTiter 96 AQueous One Solution Cell Proliferation Assay. Twenty micro-litres of MTS solution was added to 100 *μ*l of media/drug solution in each well and incubated for 60-120 minutes at 37°C. Viable cells bioreduce the MTS tetrazolium compound into a colored formazan product that is soluble in tissue culture medium. The absorbance was recorded at 490 nm with a 96-well plate reader. Background (no cells, media only) absorbance was subtracted from all other absorbances.

Absorbances from the eight wells in each lane were averaged, and the resulting absorbance value is proportional to the number of viable cells in each well of that lane/condition. Absorbance from each condition was expressed in a graph in terms of percent cell survival [compared to the absorbance of the control lane (cells-alone, no drug)]. To estimate IC_50 _(effective concentration at which 50% of the drug effects are seen) from the response curve, we apply a dose-response model that is ideal for data that has an initial response plateau, a transition region, and a final response plateau, given by(1)

for regression, where HILL is a measure of the steepness of the transition region and was fixed at a value of 2.5. *I*_*top *_is the response obtained at very low/no drug concentration and X denotes dose in logarithm unit. After regression, parameters, *I*_*top*_, *I*_*bot *_and log IC_50 _were normalized to *I*_*top *_. *I*_*bot *_is a measure of the maximum effect of the drug. A Monte Carlo simulation [43] was applied to determine the confidence level of the three fitted parameters. The idea is to simulate a bunch of data sets that are randomly sampled based on a Gaussian distribution from the ideal data set generated from the best-fit parameters. Afterward, the same regression is repeated to obtain best-fit parameters for each data set. Finally, the mean and standard deviation of best-fit parameters are determined. The log IC_50 _of cell lines exposed to twenty colchicine derivatives are listed in Table 4. Note that the use of Table 4 is only for the demonstration of the proposed approach.

**Table 4:** The log IC_50 _values of colchicine and various colchicine derivatives

	A549	HeLa	MCF-7	CEM	M010B	M006X
**D00**	-6.46 ± 0.04	-6.86 ± 0.05	-7.83 ± 0.06	-8.03 ± 0.04	-7.70 ± 0.05	-8.35 ± 0.30
**D01**	-5.29 ± 0.11	N/A	-5.25 ± 0.10	N/A	N/A	N/A
**D02**	-5.89 ± 0.06	-6.48 ± 0.13	-6.32 ± 0.11	-6.65 ± 0.08	N/A	N/A
**D03**	-5.07 ± 0.04	-5.41 ± 0.13	-5.23 ± 0.07	-5.39 ± 0.11	N/A	N/A
**D04**	-5.09 ± 0.06	-5.46 ± 0.13	-5.25 ± 0.08	-5.51 ± 0.13	-5.22 ± 0.07	-6.12 ± 0.04
**D05**	-7.83 ± 0.10	-7.89 ± 0.06	-7.42 ± 0.13	-8.64 ± 0.08	-7.94 ± 0.03	-8.41 ± 0.08
**D06**	-7.80 ± 0.07	-7.68 ± 0.09	-7.50 ± 0.13	-8.48 ± 0.09	-7.49 ± 0.13	-7.98 ± 0.03
**D07**	-7.66 ± 0.08	-8.25 ± 0.09	-8.10 ± 0.05	-8.49 ± 0.10	N/A	N/A
**D08**	-6.52 ± 0.13	-6.66 ± 0.11	-6.17 ± 0.07	-6.76 ± 0.07	-6.60 ± 0.13	-7.12 ± 0.06
**D09**	-6.66 ± 0.09	-7.35 ± 0.09	-7.10 ± 0.04	-7.46 ± 0.08	-6.74 ± 0.08	-7.47 ± 0.09
**D10**	-6.47 ± 0.12	-7.23 ± 0.09	-7.05 ± 0.04	-7.45 ± 0.09	-6.74 ± 0.08	-7.40 ± 0.09
**D11**	-5.77 ± 0.10	-6.17 ± 0.07	-6.27 ± 0.08	-6.45 ± 0.08	-5.66 ± 0.10	-6.42 ± 0.09
**D12**	-4.51 ± 0.31	-5.33 ± 0.14	-5.19 ± 0.09	-5.50 ± 0.10	-4.90 ± 0.09	-5.67 ± 0.12
**D13**	-4.95 ± 0.07	-5.33 ± 0.11	-5.22 ± 0.08	-5.53 ± 0.11	-5.20 ± 0.08	-5.44 ± 0.13
**D14**	N/A	N/A	N/A	N/A	N/A	N/A
**D15**	-6.00 ± 0.04	-6.43 ± 0.14	-6.30 ± 0.12	-6.46 ± 0.09	-6.39 ± 0.11	-6.47 ± 0.12
**D16**	-6.23 ± 0.08	-6.22 ± 0.09	-6.35 ± 0.12	-6.57 ± 0.11	-6.38 ± 0.14	-6.45 ± 0.13
**D17**	-7.38 ± 0.14	-7.77 ± 0.09	-7.37 ± 0.11	-8.47 ± 0.13	-7.65 ± 0.10	-8.34 ± 0.11
**D18**	-8.50 ± 0.12	-8.45 ± 0.11	-8.31 ± 0.13	-8.29 ± 0.10	N/A	N/A
**D19**	-8.37 ± 0.11	-8.47 ± 0.14	-8.28 ± 0.10	-8.64 ± 0.09	-8.27 ± 0.12	-8.83 ± 0.08
**D20**	-8.76 ± 0.10	-8.66 ± 0.13	-8.71 ± 0.11	-8.55 ± 0.09	-8.51 ± 0.14	N/A

The values are determined by cytotoxicity testing on six different cell lines. Each IC_50 _value represents the Effective Concentration at which the drug shows 50% of its efficacy on each particular cell line.

### Tubulin Isotype expression data

Following cytotoxicity assays which gave us an indication that the designed colchicine derivatives have led to encouraging outcomes for a panel of diverse cancer cell lines, our next task was to determine the expression levels of tubulin isotypes before and after exposure of the cells to the toxic agents including colchicines and D20 at the estimated 25% of lethal concentration of dose. This type of experiment would provide information regarding the regulation by the cells of the target protein, tubulin, and its specific isoforms.

Briefly, frozen cell pellets from six human cell lines A549, MCF7, CEM, HeLa, M006X, M010B were thawed on ice and homogenized on ice in 10 mM Tris pH 7.4 and 0.3% SDS with 1% protease and phosphatase inhibitors (Sigma Chemical Co., St. Louis, MO) and centrifuged at 12,000 × g at 4°C. The supernatants were treated with 50 *μ*g/ml DNase and 100 *μ*g/ml RNase. Protein was quantified using Micro BCA protein assay kit (Pierce Biotechnology, Rockford, IL). An equal amount of protein (20 *μ*g) was boiled in SDS loading buffer containing 0.1 M DTT and resolved by electrophoresis in 7.5% SDS polyacrylamide gels (Bio-Rad Laboratories, Hercules, CA). Biotinylated protein ladder (Cell Signaling Technology, Beverly, MA) and Kaleidoscope pre-stained protein standard (Bio-Rad Laboratories, Hercules, CA) were included for assessment of molecular mass of target proteins. The gels were transblotted to Hybond-C nitrocellulose membranes (Amersham Biosciences, Piscataway, NJ) and the membranes were blocked in 5% ECL Advance blocking solution (Amersham Biosciences, Piscataway, NJ) for 1 h at room temperature. Immunodetection was performed by incubating the membrane overnight at 4°C with murine monoclonal antibodies to *β *tubulin isotypes I through IV with murine monoclonal antibodies to *β *tubulin isotypes I through IV or with a murine monoclonal antibody to *β*-actin as a housekeeping gene product control (Oncogene Research Products, Boston, MA) followed by incubation with the secondary antibody, anti-mouse IgG HRP conjugate (Cell Signaling Technology, Beverly, MA). Antibody dilutions were as follows: *β*I (1:5000, 2nd antibody 1:5000), *β*II (1:5000, 2nd antibody 1:1000), *β*III (1:5000, 2nd antibody 1:1000), and *β*IV (1:5000, 2nd antibody 1:500). The secondary antibody solution also included 1:10,000 anti-biotin HRP-linked antibody to visualize the protein ladder. All antibodies were diluted with 2.5% ECL Advance blocking solution. Target proteins were visualized by enhanced chemiluminescence with ECL Advance Western Blot detection kit and captured on Hyperfilm-ECL film (both from Amersham Biosciences). Image analysis was performed with a personal densitometer (Amersham Biosciences). Relative quantities of target proteins were determined using ImageQuant and normalized to *β*-actin levels in each sample. Furthermore, we normalized it across four isotypes. The data are shown in Table 5.

**Table 5:** Experimental tubulin isotype expression level in five cell lines

	Treatment	I	II	III	IV
**A549**	Normal	0.264	0.268	0.462	0.006
	Colchicine	0.316	0.267	0.414	0.002
	D20	0.188	0.36	0.429	0.024
**HeLa**	Normal	0.452	0.139	0.352	0.057
	Colchicine	0.436	0.138	0.37	0.055
	D20	0.395	0.227	0.258	0.119
**MCF-7**	Normal	0.112	0.596	0.291	0
	Colchicine	0.168	0.499	0.333	0
	D20	0.162	0.309	0.509	0.02
**CEM**	Normal	0.279	0.16	0.56	0
	Colchicine	0.852	0	0	0.148
	D20	0.328	0.181	0.481	0.009
**M010B**	Normal	0.393	0.24	0.329	0.038
	Colchicine	0.446	0.19	0.259	0.106
	D20	0.466	0.189	0.243	0.101

### The maximum entropy method for tubulin Isotype expression level estimates

#### Maximum entropy method: a tool for assigning and updating probability distributions

Our goal was to utilize information such as binding free energy values between a toxic agent and a molecular target in order to estimate tubulin isotype expression levels present in cytotoxcity assays. This is exactly the type of question that the method of maximum entropy is designed to answer [28-34]. Specifically, based on the assumption that cytotoxicity is correlated with drug affinity for the molecular target, we ask: "G*iven the information regarding the binding free energy between individual tubulin isotypes and colchicine derivatives and the IC _50 _values from cytotoxic measurements on the cell lines exposed to these drugs, what are most likely expression levels of specific tubulin isotypes?*".

Following Jaynes' method of maximum entropy (MaxEnt) [29], which is only designed to codify limited information relevant to systems of interest into a probability distribution with the least bias, the ME method [30-35] is designed to update the corresponding probability distribution from an *a priori *chosen function each time additional information is acquired. Note that the probability distribution represents our state of knowledge of the systems in a specific state. Suppose the system of interest is characterized by label i and the variable *H*_*i *_represents some properties of the system in state i. For example, the label i may represent a binding mode of target proteins and ligands. Furthermore, suppose that probability values *μ*_*i *_are given as priors before any measurements are performed. Suppose also the new information *H *in the form of(2)

is acquired. The preferred *P*_*i *_that codifies this new information *H *and the prior is *μ*_*i *_determined by maximizing the relative entropy given by(3)

subject to the normalization constraint and the constraint in Eq. (2). The preferred posterior probability is then given by,(4)

where the partition function is defined in a standard way as  and the Lagrange multiplier *β *can be determined by substituting Eq. (4) back into Eq. (2).

#### Information relevant to the expression level estimate

The method of ME provides a robust and universal route for information processing with the least bias. The ME method ensures that the ME posterior probability distribution is preferred over any other probability distributions used to characterize the system of interest as long as the information used to update from a prior is relevant to the system. Thus, to estimate the expression levels of tubulin isotypes in cytotoxicity assays involving the novel colchicine derivatives, we only need to focus on determining the information that is relevant to the interactions between tubulin isotypes and colchicine derivatives and the relationship with the outcomes of the cytotoxicity assays.

The interaction between tubulin isotypes and colchicine derivatives (colchicine is denoted by C and its derivatives are collectively denoted by C') is best described by the binding free energy of tubulin isotypes and either C or C'. The total binding free energy in each cell line is the average of binding free energy of M tubulin isotypes Δ*G*_*i *_with specific weighting factors *P*_*i *_representing relative expression levels of individual isotypes,(5)

where subscript i labels the type of tubulin isotype. Based on the ME method, ⟨Δ*G*⟩ if can be measured, then the preferred weighting or the expression level can be determined.

Although it is difficult to directly measure the binding free energy for the entire cell line, the information regarding the interaction between tubulin isotypes and colchicine derivatives can still be described by the cytotoxicity measurement of the values of log IC_50_. The studies of Tian and Haffner show a linear relation between cytotoxicity-derived values of logIC_50 _and the binding free energy [44],(6)

Even though the total binding free energy can be estimated from log IC_50 _as clearly seen from Eq. (6), we still require the individual contribution values towards the total binding free energy coming from tubulin isotypes Δ*G*_*i *_for each of the administered colchicine derivatives. Because of the complicated conformational changes in the physical binding processes, calculations of the binding free energy of tubulin isotypes i and colchicine  or colchicine derivatives  is a challenging and demanding computational task to perform. However, we have succeeded in this as has been reported in Sec.1. These results will be used in the application of the ME method as input data, along with the IC_50 _values obtained from cytotoxicity measurements.

Finally, having estimated ⟨Δ*G*⟩ from log IC_50 _using Δ*G*_*i *_estimates from ΔΔ*G*_bind_*i*_, we can now re-formulate the constraint equation that relates the cytotoxicity of colchicine derivatives given by the values of log IC_50 _and the binding free energy between tubulin isotypes and colchicine derivatives as(7)

where we use superscript *α *= C or C' to denote the cell line exposed to colchicine or colchicine derivatives, respectively.

#### ME static expression level

The ME method gives the probability distribution that is updated from a prior *μ*_*i *_with the information given by Eq. (7) as(8)

where the partition function  and the coefficient *β*^*α *^is determined from Eq. (7). This will be used as the normalized expression level of tubulin isotype i in the cell line exposed to colchicine derivative *α*. However, because  cannot be calculated directly, this expression level cannot be determined yet. Fortunately, we can apply the ME again to resolve this issue. Because our goal is to study the cytotoxicity of colchicine derivatives and compare it to the case when the cells are exposed to standard colchicine, we can utilize later studies as a prior information. Suppose the tubulin isotype expression level when a cell line is exposed to colchicines is given by . We then can rewrite Eq. (8) as(9)

where *g*_*i *_is a dummy variable and we set *μ*_*i *_= 1 to indicate that no other prior information is included. Furthermore, the constraint, Eq. (7) can be rewritten as(10)

Therefore, the binding free energy involving a tubulin isotype and colchicine can be estimated from  through Eqs. (9) and (10). Next, since , the binding free energy involving a tubulin isotype and colchicine derivatives is . Therefore, we can determine the expression level of tubulin isotype i in cell lines exposed to colcichine-based derivatives by(11)

where we consider the normalized expression level  as the prior *μ*_*i*_. Furthermore, the practically of Eq. (11) requires an initial guess of *β*^*C' *^in order to solve Eq. (7). In our studies, in order to generate a statistically relevant data set, we will repeat the same calculation one hundred times with various initial guesses for *β*^*C' *^generated by random selection. Both the mean value *β*_*C' *_and its standard deviation will be used in our calculations.

## Results and Discussion

### Validation and the effects of different prior information

To validate the proposed approach, we consider the cell lines used except M006X and one colchicine derivative D20 as the benchmark because of the sufficient experimental expression data for these five cell lines exposed to D20 (see Table 5). We also investigate the effects of different prior information required in the proposed approach. Because the tubulin isotypes *αβ*IIa and *αβ*IIb are structurally indistinguishable in some experiments, as are the isotypes *αβ*IVa and *αβ*IVb, we average relative binding energy of isotypes *αβ*IIa and *αβ*IIb and isotypes *αβ*IVa and *αβ*IVb as isotypes *αβ*II and *αβ*IV, respectively, to account for this property.

#### Results for no experimental expression data available

**Figure 4:**
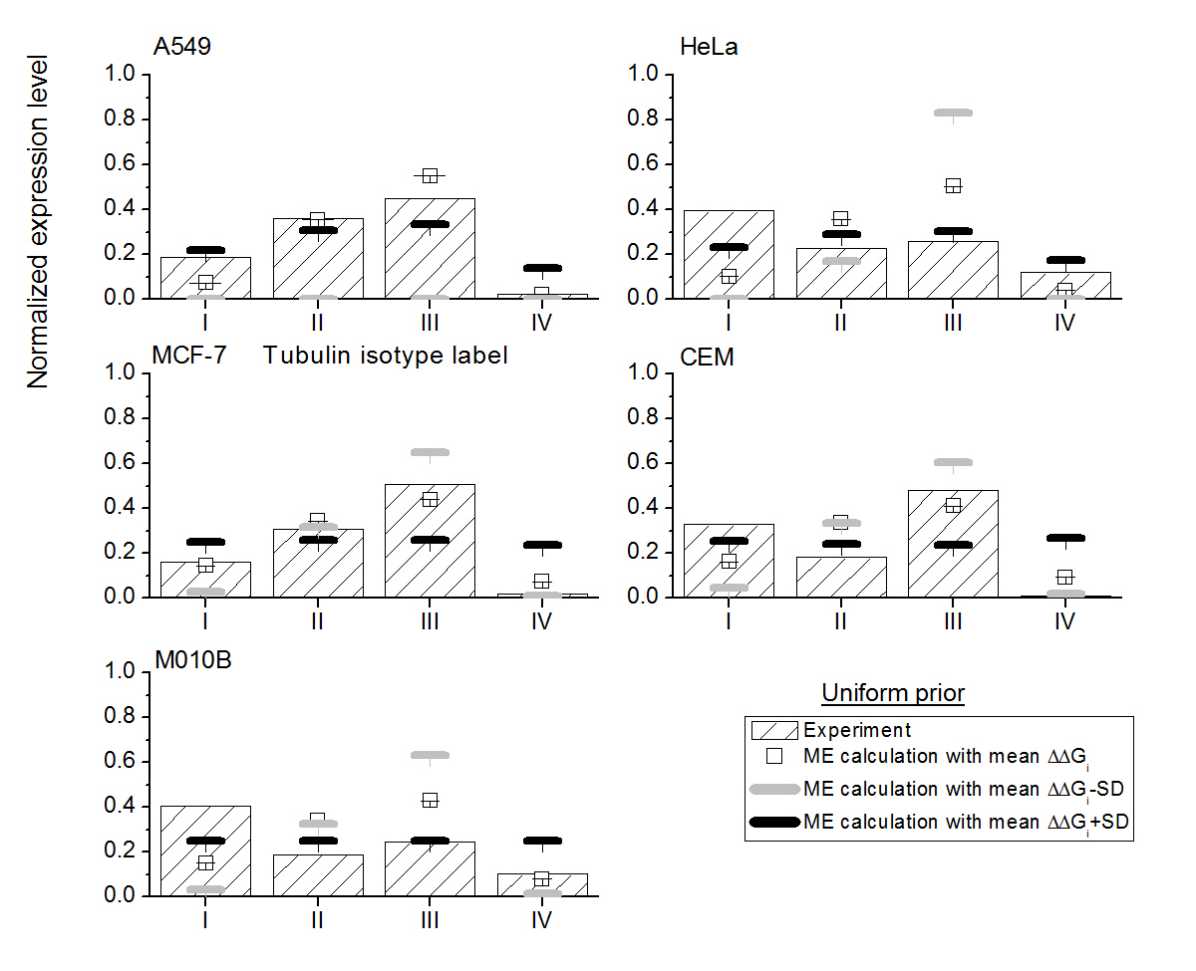
**The ME expression levels with the consideration of a uniform prior **The ME expression levels of four tubulin isotypes in five cell lines exposed to D20 with the consideration of a uniform prior. The hollow square denotes the ME expression level calculated based on mean relative binding free energy ΔΔ*G*_bind_*i*_. The black and gray bar are the ME expression level calculated based on mean ΔΔ*G*_bind_*i *_± its standard deviation (SD). The histogram with sparse oblique strips denotes experimental expression level data.

Suppose there is not enough experimental expression of tubulin isotypes in cell lines exposed to colchicine, the optimal choice then is to assume that the five tubulin isotypes are equally expressed in cell lines based on Bernoulli's principle of insufficient reason [45]. Therefore, we propose to set a uniform prior, , where M denotes the total number of tubulin isotypes in the proposed approach. We can then estimate the expression level of tubulin isotypes in five cell lines with D20 present from Eqs. (9), (10) and (11). Figure 4 shows the ME results and compares them to the experimental measurement values denoted by histogram. The open squares are calculated using the mean relative binding free energy and will be taken to represent the mean ME expression level of tubulin isotypes. The dark and light gray bars are obtained using mean plus and minus standard deviation (SD) of relative binding free energy, respectively, and they will be noted as mean ± SD ME expression level.

The ME predictions based on mean relative binding free energy values and mean plus and minus standard deviations all give roughly the same trends for all five cell lines. However, this only agrees with the experimental observations for the cell line A549, MCF-7 and CEM. We believe that the reason for this finding is that the expression levels of tubulin isotypes in cell lines exposed to colchicine except A549, MCF-7 and CEM are dramatically different from the uniform prior, which was taken to represent uniform expression levels for all tubulin isotypes. For the case of no prior information including in the calculation, these results are simply resulted from that D20 and isotype *αβ*III has the largest relative binding free energy.

#### Results for the case with limited experimental expression data available

**Figure 5:**
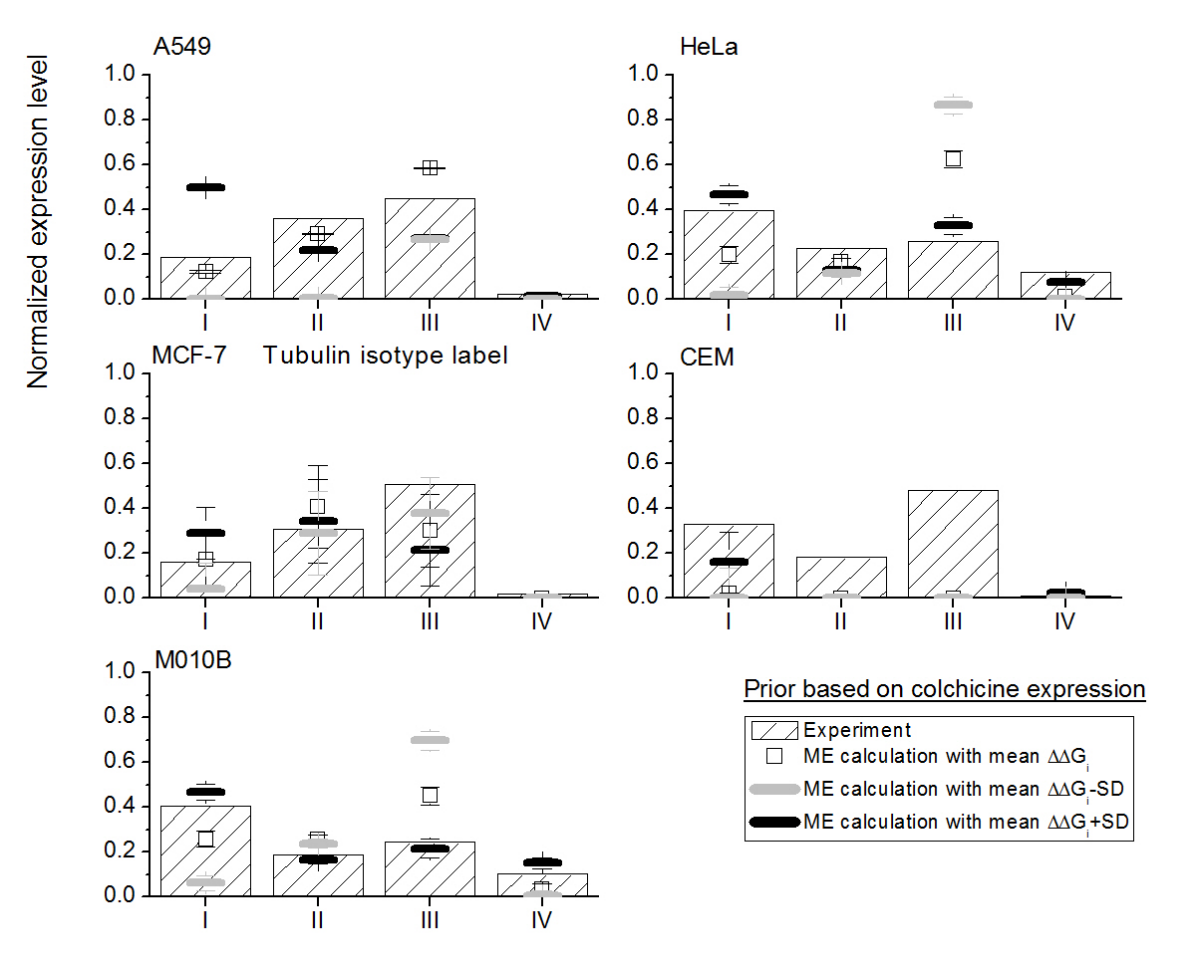
**The ME expression levels with the consideration of prior estimated from colchicine data**. ME expression levels of four tubulin isotypes in five cell lines exposed to D20 with the consideration of prior estimated from experimental data for colchicine. The hollow square denotes the ME expression level calculated based on mean relative binding free energy ΔΔ*G*_bind_*i*_. The black and gray bar are the ME expression level calculated based on mean ΔΔ*G*_bind_*i *_± its standard deviation (SD). The histogram with sparse oblique strips denotes experimental expression level data.

Whenever the expression data for tubulin isotypes in cell lines exposed to colchicine is available, we considered the normalized expression level as the prior . Therefore, having obtained the expression data measured in five cell lines exposed to D20, the ME calculations are shown in figure 5 with the same definitions of symbols as those in figure 4. This figure shows several key features emerging from ME calculations. First, in A549 and MCF-7, the figure shows the experimental observations are almost distributed within the mean ± SD ME expression levels again. Second, for HeLa and M006B, the mean-SD ME expression levels are almost coincide with the experimental observations.

It should be emphasized that the choice of a "good" prior and accurate binding free energy estimates are crucial. As shown in both cases, when the prior takes into account the experimental expression data and appropriate relative binding free energy, the proposed approach is likely to give correct tubulin expression levels. However, one should not dismiss the use of the uniform prior. One can expect the ME calculation with the uniform prior to predict the expression levels similar to experimental observations when the variations of actual expression levels for tubulin isotypes in cell lines exposed to colchicine are not dramatically differed from the uniform distribution. Cell line A549 and MCF-7 are excellent examples of this type of outcome.

Next, we will utilize the prior assigned from experimental data to further investigate two questions, namely: "*What are the expression levels of tubulin isotypes in cell lines exposed to various colchicine derivatives?*" and "*What are the predicted tubulin isotype expression levels when the similar isotype pairs: αβIIa and αβIIb, and αβIVa and αβIVb are distinguishable in cell lines with the presence of different derivatives?*"

### ME expression levels of tubulin isotypes when cell lines are exposed to all derivatives

#### Case 1

Tubulin isotype *αβ*IIa and *αβ*IIb as well as *αβ*IVa and *αβ*IVb are considered to be structurally indistinguishable

### Tubulin isotype *αβ*III is a primary target of colchicine derivatives D01, D02, D03, D04, D05, D06, D18 and D20

**Figure 6:**
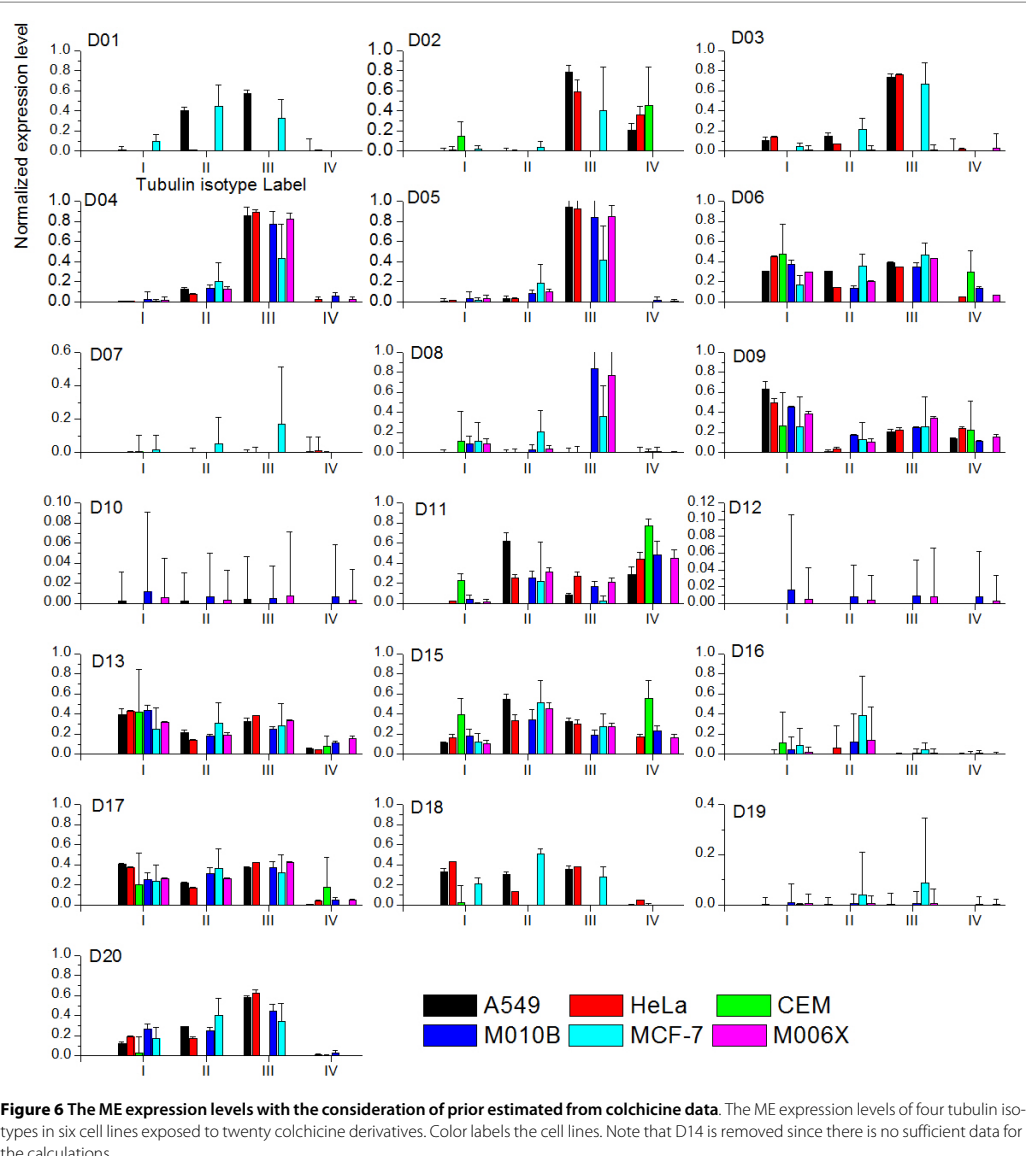
**The ME expression levels with the consideration of prior estimated from colchicine data**. The ME expression levels of four tubulin isotypes in six cell lines exposed to twenty colchicine derivatives. Color labels the cell lines. Note that D14 is removed since there is no sufficient data for the calculations.

All six cell lines are studied given the tubulin isotypes expression levels in each cell line exposed to colchicines as a prior. When the ME predicted expression level for a given colchicine derivative cannot be determined, zero expression levels are assigned. One can attribute it to the fact that relative binding free energy given such a derivative is ill defined. There is no solution for Eq. (7). The results of our analysis are plotted in figure 6.

**Figure 7:**
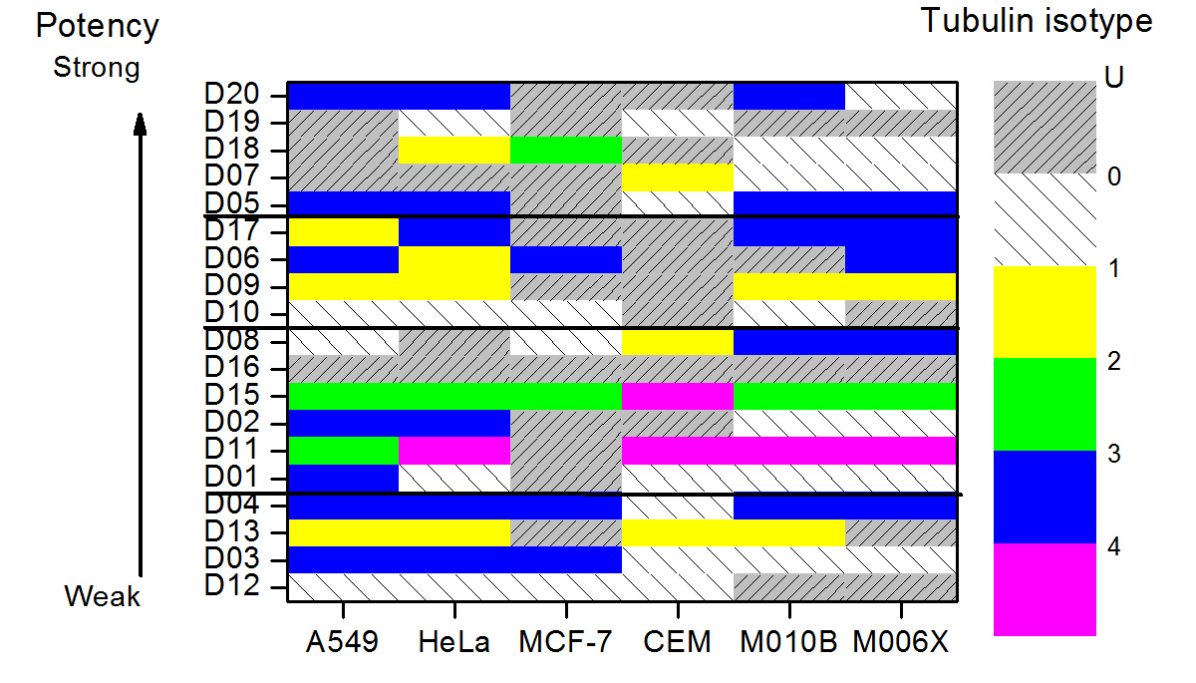
**Plot of colchicine derivatives vs. cell lines**. The distribution of tubulin isotypes with the highest expression levels in six cell lines, where they are exposed to 20 colchicine derivatives. The color map level 1 to 5 indicates the types of tubulin isotypes. Label "U" denotes that there are multiple isotypes that have the same highest expression level. Label "0" denotes no ME prediction can be made. All the colchicine derivatives are sorted in the order of potency (logIC_50 _value).

We further summarize and illustrate the tubulin isotype distribution with the highest expression levels in cell lines exposed to the twenty colchicine derivatives in figure 7 to investigate the effects of the colchicine derivatives on the expression levels of tubulin. Note that the colchicine derivatives are plotted in the order of potency from weak at bottom toward strong at top based on the corresponding IC_50 _values. The remaining labels show the same order of tubulin isotype expression. The figure shows several important features. First, tubulin isotype *αβ*III is likely to show the highest expression level for the cell line A549, Hela, M010B and M006X exposed to the colchicine derivatives D03, D04, D05, and D20. For MCF-7, it has the highest expression level for the colchicine derivatives D03, D04, and D06. Second, for colchicine derivatives D09 and D13, Figure 7 shows that isotype *αβ*I has the highest expression level in all cell lines except MCF-7 and CEM. Furthermore, it also has the highest level for cell line CEM with three out of five derivatives. Third, for cell line MCF-7, it is either isotype *αβ*II or *αβ*III that has the highest expression level. Finally, the frequency score of the isotypes with the highest expression level is 13, 7, 26, 5, 35 and 28 for the tubulin isotypes *αβ*I, *αβ*II, *αβ*III, *αβ*IV, labelled "U" and "0", respectively. Isotype *αβ*III is ranked first and is followed by *αβ*I and *αβ*II over all. Fourth, Figure 5 shows no obvious correlation between the potency of derivatives and the isotypes with the highest expression level, particularly, *αβ*III, the potency is unlikely a factor to influence the binding affinity. Finally, this suggests that tubulin isotypes *αβ*III followed by *αβ*I and *αβ*II are the first three primary targets of colchicine derivatives in all cell lines except CEM giving us important insights for future optimization of drug design based on colchicine derivatives.

#### Case 2

Only tubulin isotype *αβ*IVa and *αβ*IVb are structurally distinguishable

### Both isotype *αβ*IVa and IVb have relatively low expression levels

**Figure 8:**
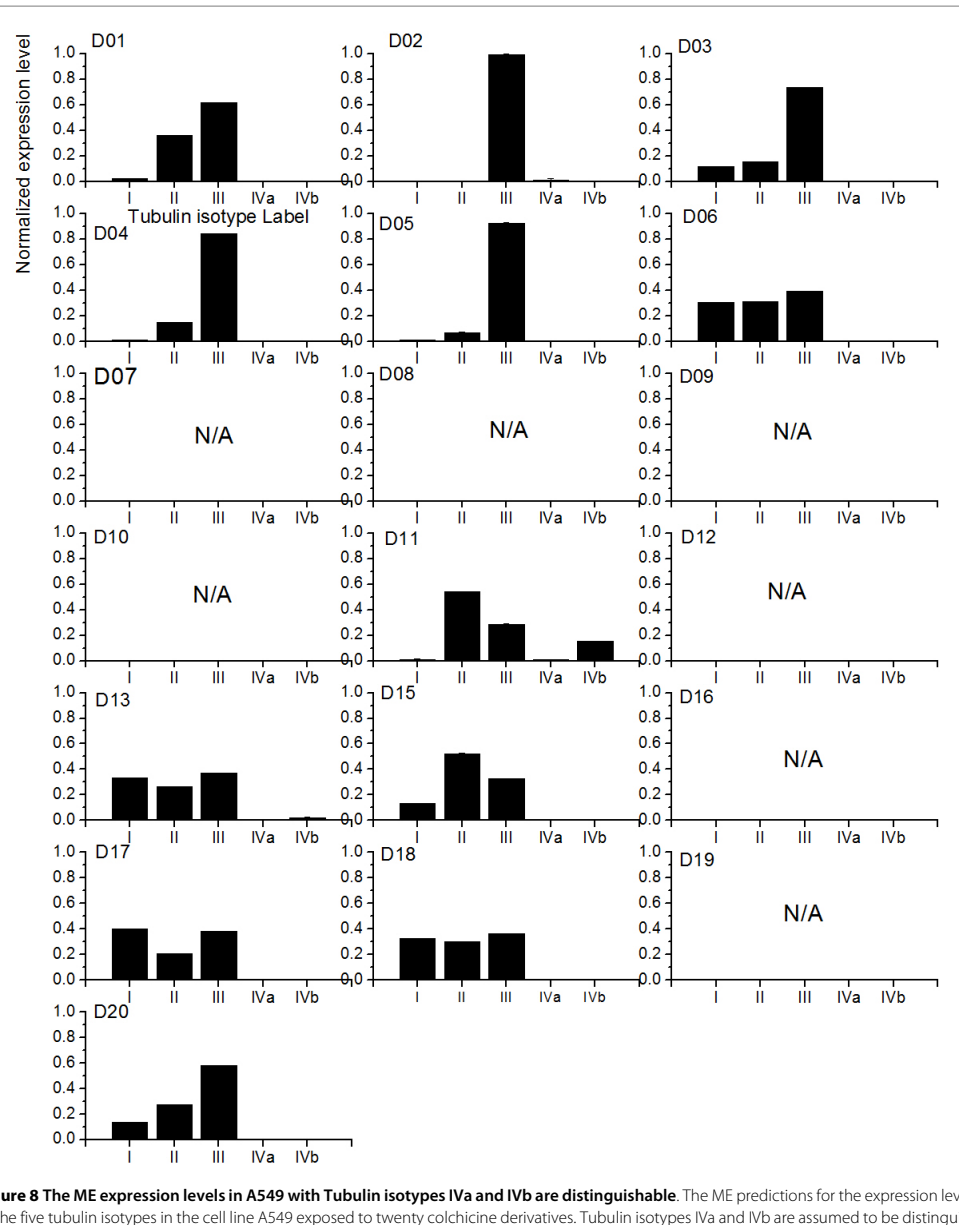
**The ME expression levels in A549 with Tubulin isotypes IVa and IVb are distinguishable**. The ME predictions for the expression levels of the five tubulin isotypes in the cell line A549 exposed to twenty colchicine derivatives. Tubulin isotypes IVa and IVb are assumed to be distinguishable. Note that N/A indicates no solution can be found.

Next, we study the application of ME method to the case when tubulin isotype *αβ*IVa and *αβ*IVb are structurally distinguishable in cell lines. Because experimental measurements on expression levels are only available for the isotype *αβ*IV, we simply assign both *αβ*IVa and IVb with the same prior, which is half of the normalized expression level of isotype *αβ*IV for the calculations. Furthermore, we only take A549 cell line as an example for our investigations (see figure 8). In general, the difference between isotype *αβ*IVa and *αβ*IVb does not influence the trend of expression levels for the case when tubulin isotypes *αβ*IVa and *αβ*IVb are indistinguishable. There is only one case using D09 that shows results opposite to the one from figure 6. We therefore concluded that there is no solution for this particular case.

In addition, there is no major difference between both isotypes with both having relatively low expression levels for cell line A549 exposed to all colchicine derivatives, yet for derivative D11, the isotype *αβ*IVb has a higher expression level than tubulin *αβ*IVa.

#### Case 3

All tubulin isotypes are structurally distinguishable

### Tubulin isotype *αβ*III is the primary target for twelve out of twenty colchicine derivatives

**Figure 9:**
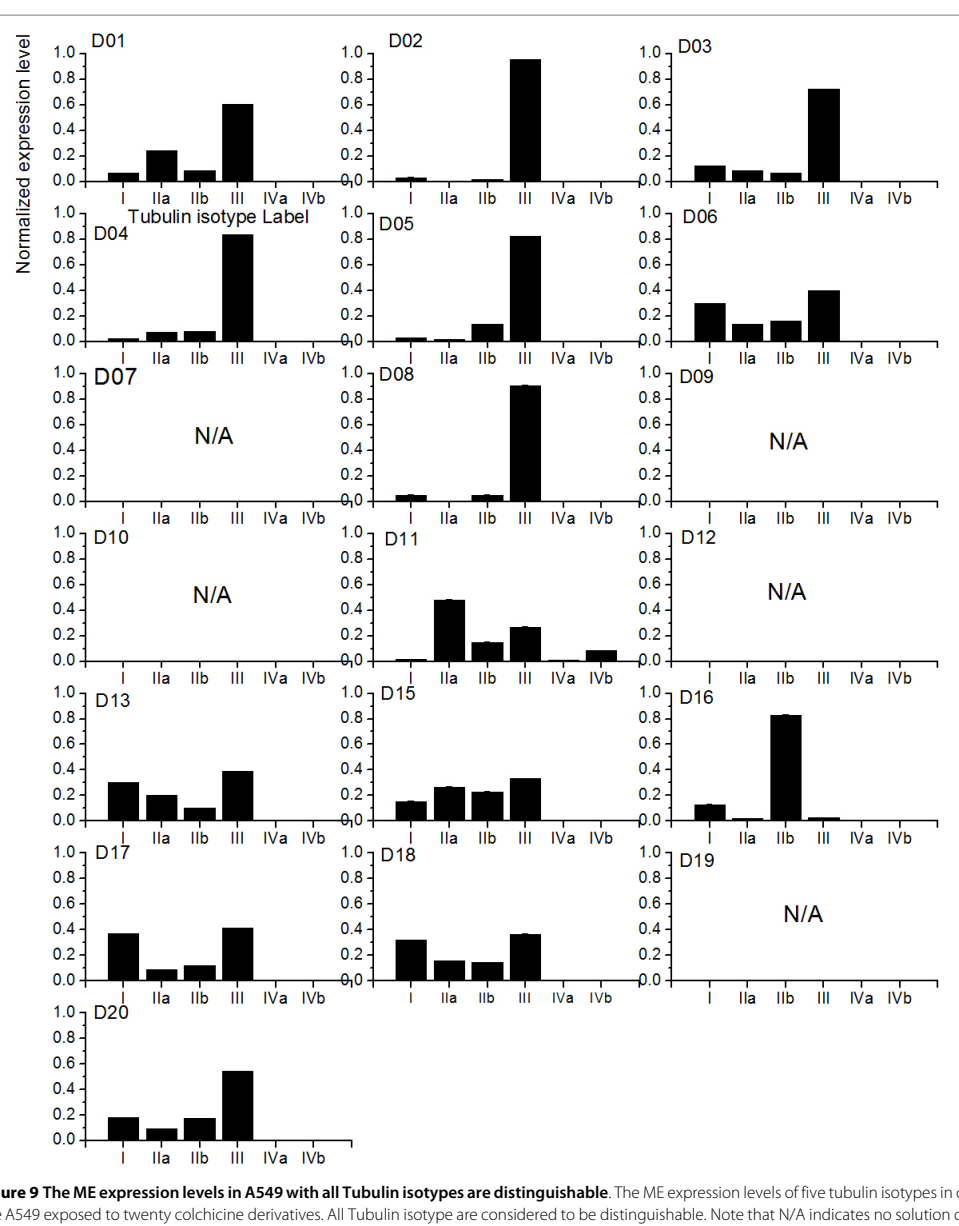
**The ME expression levels in A549 with all Tubulin isotypes are distinguishable**. The ME expression levels of five tubulin isotypes in cell line A549 exposed to twenty colchicine derivatives. All Tubulin isotype are considered to be distinguishable. Note that N/A indicates no solution can be found.

We then investigated the effects when all tubulin isotypes are structurally distinguishable in the cell line A549. In general, when we take the differences between tubulin isotypes *αβ*IIa and *αβ*IIb in addition to *αβ*IVa and *αβ*IVb into account, the ME calculation shows that the scorefor the tubulin isotype *αβ*III when it has the highest expression level in the cell line A549 is increased from 8 to 13 (see figure 9). This observation suggested that tubulin isotype *αβ*III is a primary target for twelve out of twenty derivatives.

### The expression level of tubulin isotype *αβ*IIa and *αβ*IIb shows two trends

For D01, 11, 13 and 15, the ME calculation shows that tubulin isotype *αβ*IIa has a higher expression level than *αβ*IIb. However, when A549 is exposed to D05, D08, D16, D17 and D20, the calculation shows the opposite trend that isotype *αβ*IIb is expressed over *αβ*IIa. For the rest of derivatives D03, 04, 06, D18, both roughly have the same expression level.

We have applied the maximum entropy approach to predict the tubulin isotype expression levels to study cytotoxic effects due to the binding of tubulin isotypes in several human cancer cell lines subjected to a panel of colchicine derivatives. Experimental assays leading to this report and performed by us include cytotoxicity on cancer cell lines for each of the 20 drug molecules from the same family of colchicine compounds. The tubulin isotype expression level measurements were performed under three conditions in the cell lines, which received no treatment (normal), were exposed to colchicine and were exposed to the compound D20. The maximum entropy method is used in conjunction with binding energy calculations for the colchicine derivatives, as they were found to interact specifically with tubulin isotypes. We found that depending on the use of assumptions regarding the priors in the method applied to the probability distribution functions, various outcomes may be obtained. However, in the presence of experimentally available information, a much more consistent picture emerges. Most importantly, in almost all cases studied we have identified the tubulin isotype *αβ*III as the first most important molecular target for the action of the colchicine derivatives that inhibit the polymerization of MTs. We have also found the isotypes *αβ*I and *αβ*II as secondary targets but their significance is substantially diminished due to the widespread expression throughout the human body, especially true for *αβ*I, in contrast to *αβ*III which is limited to a few specific tissues and cancer cells.

## Conclusions

We have demonstrated the applicability of the maximum entropy approach in predicting cytotoxic effects based on limited information such as the relative binding energy values for the cytotoxic agents used. Namely, given the relative binding free energy of tubulin isotype and colchicine derivatives, the proposed approach predicts the tubulin isotype expression levels in various cell lines exposed to colchicine derivatives. Our studies also provide a better defined molecular target for the action of these anti-mitotic drugs, namely, tubulin isotype *αβ*III, for optimized chemotherapy drug design compared to earlier efforts in this area. By narrowing down the focus of tubulin targets to this isotype, most dramatically regulated by cancer cells when exposed to the colchicine derivatives, both the efficacy and specificity of treatment will hopefully be improved.

Unfortunately, the currently used chemotherapy drugs do not particularly target *αβ*III tubulin isotype, hence the best compounds revealed in this study may offer a potential improvement in clinical outcomes. An interesting question to ask is why the currently used compounds show good efficacies without specifically targeting tubulin isotype *αβ*III. The probable reason why taxanes and vinblastine are successful is that they preferentially interact with the *αβ*II isotype [3,46] which is widespread in cancers [47], but has a somewhat limited distribution in normal cells, being found largely in nerves and at low levels elsewhere [19,48]. Not surprisingly, these drugs cause neurotoxicity [49-51]. In contrast, the parent compound colchicine prefers to bind to *αβ*IV, an isotype widespread in normal tissues that is not common in cancers [7]. Hence, prolonged chemotherapy with colchicines would not be useful due to severe side effects such as kidney and liver toxicity.

The *αβ*III isotype of tubulin singled out in the present paper is an almost perfect target for breast cancer chemotherapy because a) it is found in many tumors, especially those that are metastatic and aggressive, including breast tumors [21,52-55]; and b) its normal distribution is even more limited than that of *αβ*II, occurring largely in the brain and the testes [19]. *αβ*III accounts for only 25% of brain *αβ*-tubulin while *αβ*II constitutes 58% [56] suggesting a reduced neurotoxicity [57]. Furthermore, while *αβ*II is found in both neurons and glial cells, *αβ*III occurs only in neurons [48].

Other researchers have already realized that *αβ*III would be an excellent target for anti-tumor drugs [58] and have attempted to use rational drug design to create a *αβ*III-specific drug. The seco-taxoid, IDN 5390 [25,59] was explicitly designed to bind to the taxane binding site on *αβ*III. This drug is very effective against paclitaxel-resistant cell lines over-expressing *αβ*III. The epothilone derivative ixabepilone which is very effective against human cancer cell lines and xenograft models that over-express *αβ*III and under-express *αβ*II, is now recommended for use with metastatic taxane-resistant or taxane-untreated breast cancers [60,61]. However, despite being apparently very promising, these new drugs may have some serious limitations. First, it has now been shown experimentally *in vitro *that either ixabepilone or IDN 5390 binds well to *αβ*III and poorly to other isotypes. The *in vivo *data indeed indicate that these compounds target *αβ*III [59,60] but do not rule out their strong binding to other isotypes. Second, the observation that ixabepilone has the same toxicity profile as paclitaxel, including neuropathy [61,62], and the fact that IDN 5390 is recommended to be used in conjunction with taxanes [25] indicates that the neurotoxicity issue is still not resolved. Third, it may be that the fundamental problem with the process that led to the design of these drugs is that no effort was made to figure out the actual physiological function of their intended target: the *αβ*III isotype---nor of the targets to be avoided, such as the *αβ*I or *αβ*II isotypes.

On the other hand, our novel colchicine derivatives bind at a different site on tubulin than do either IDN 5390 or ixabepilone. Thus, we are exploring a new area here. There is no reason to expect these derivatives to have the same toxicity and side effects as does colchicine, the parent compound. Since its toxicity arises from the fact that standard colchicine has a very high affinity for the widespread *αβ*IV isotype, while it binds very poorly to *αβ*III. These novel compounds were designed to bind well to *αβ*III and hence should not cause the same type of toxicity as does colchicines.

## Competing interests

The authors declare that they have no competing interests.

## Authors' contributions

CYT carried out the development of maximum entropy approach, performed tubulin expression level analyses and drafted the manuscript. JM carried out the QM/MM binding affinity calculation. TH was involved in the preparation of tubulin isotype structures for binding affinity calculations and the analysis of binding modes for colchicine derivatives. PW carried out cytotoxicity statistical analysis. LJ performed all cytotoxicity experiments. EI carried out the essential work required for the tubulin isotype expression assays. RFL contributed to the identification of specific tubulin isotypes as chemotherapy targets and participated in tubulin expression assays. JAT conceived of the study, and participated in its design and coordination and helped to draft the manuscript. All authors read and approved the final manuscript.

## References

[B1] Fellous A Francon J Lennon AM Nunez J Microtubule assembly in vitro. Purification of assembly-promoting factors Eur J Biochem 1977 78 167 174 10.1111/j.1432-1033.1977.tb11726.x 913395

[B2] Owellen RJ Owens AHJ Donigian DW The binding of vincristine, vinblastine and colchicine to tubulin Biochem Biophys Res Commun 1972 47 685 691 10.1016/0006-291X(72)90546-3 5026289

[B3] Derry WB Wilson L Khan IA Luduena RF Jordan MA Taxol differentially modulates the dynamics of microtubules assembled from unfractionated and purified beta-tubulin isotypes Biochemistry 1997 36 3554 3562 10.1021/bi962724m 9132006

[B4] Nogales E Wolf SG Khan IA Luduena RF Downing KH Structure of tubulin at 6.5 A and location of the taxol-binding site Nature 1995 375 424 427 10.1038/375424a0 7760939

[B5] Lowe J Li H Downing KH Nogales E Refined structure of alpha beta-tubulin at 3.5 A resolution J Mol Biol 2001 313 1045 1057 10.1006/jmbi.2001.5077 11700061

[B6] Weick JK Livingston RB Van Slyck EJ Colchicine in refractory chronic lymphocytic leukemia Invest New Drugs 1983 1 335 338 6678880 10.1007/BF00177418

[B7] Banerjee A Luduena RF Kinetics of colchicine binding to purified beta-tubulin isotypes from bovine brain J Biol Chem 1992 267 13335 13339 1618835

[B8] Ravelli RB Gigant B Curmi PA Insight into tubulin regulation from a complex with colchicine and a stathmin-like domain Nature 2004 428 198 202 10.1038/nature02393 15014504

[B9] Tahir SK Kovar P Rosenberg SH Ng SC Rapid colchicine competition-binding scintillation proximity assay using biotin-labeled tubulin Biotechniques 2000 29 156 160 10907090 10.2144/00291rr02

[B10] Russell GJ Lacey E Inhibition of [3H]mebendazole binding to tubulin by structurally diverse microtubule inhibitors which interact at the colchicine binding site Biochem Mol Biol Int 1995 35 1153 1159 7492951

[B11] Garland DL Kinetics and mechanism of colchicine binding to tubulin: evidence for ligand induced conformational change Biochemistry 1978 17 4266 4272 10.1021/bi00613a024 708711

[B12] Sackett DL Varma JK Molecular mechanism of colchicine action: induced local unfolding of beta-tubulin Biochemistry 1993 32 13560 13565 10.1021/bi00212a023 8257691

[B13] Andreu JM Timasheff SN Conformational states of tubulin liganded to colchicine, tropolone methyl ether, and podophyllotoxin Biochemistry 1982 21 6465 6476 10.1021/bi00268a023 7150568

[B14] Chaudhuri AR Seetharamalu P Schwarz PM Hausheer FH Luduena RF The interaction of the B-ring of colchicine with alpha-tubulin: a novel footprinting approach J Mol Biol 2000 303 679 692 10.1006/jmbi.2000.4156 11061968

[B15] Dumont R Brossi A Chignell CF Quinn FR Suffness M A novel synthesis of colchicide and analogs from thiocolchicine and congeners: reevaluation of colchicide as a potential antitumor agent J Med Chem 1987 30 732 735 10.1021/jm00387a028 3560165

[B16] Brossi A Yeh HJ Chrzanowska M Wolff J Hamel E Lin CM Quin F Suffness M Silverton J Colchicine and its analogs: recent findings Med Res Rev 1988 8 77 94 10.1002/med.2610080105 3278182

[B17] Huzil JT Luduena RF Tuszynski J Comparative modelling of human beta-tubulin isotypes and implications for drug binding Nanotechnology 2006 17 S90 S100 10.1088/0957-4484/17/4/014 21727360

[B18] Lu Q Luduena RF In vitro analysis of microtubule assembly of isotypically pure tubulin dimers. Intrinsic differences in the assembly properties of alpha beta II, alpha beta III, and alpha beta IV tubulin dimers in the absence of microtubule-associated proteins J Biol Chem 1994 269 2041 2047 8294455

[B19] Luduena RF Multiple forms of tubulin: different gene products and covalent modifications Int Rev Cytol 1998 178 207 275 10.1016/S0074-7696(08)62138-5 9348671

[B20] Roach MC Boucher VL Walss C Ravdin PM Luduena RF Preparation of a monoclonal antibody specific for the class I isotype of beta-tubulin: the beta isotypes of tubulin differ in their cellular distributions within human tissues Cell Motil Cytoskeleton 1998 39 273 285 10.1002/(SICI)1097-0169(1998)39:4<273::AID-CM3>3.0.CO;2-4 9580378

[B21] Katsetos CD Legido A Perentes E Mork SJ Class III beta-tubulin isotype: a key cytoskeletal protein at the crossroads of developmental neurobiology and tumor neuropathology J Child Neurol 2003 18 851 66 discussion 867. 10.1177/088307380301801205 14736079

[B22] Katsetos CD Kontogeorgos G Geddes JF Differential distribution of the neuronassociated class III beta-tubulin in neuroendocrine lung tumors Arch Pathol Lab Med 2000 124 535 544 10747310 10.5858/2000-124-0535-DDOTNA

[B23] Scott CA Walker CC Neal DA Beta-tubulin epitope expression in normal and malignant epithelial cells Arch Otolaryngol Head Neck Surg 1990 116 583 589 1691648 10.1001/archotol.1990.01870050083012

[B24] Banerjee A Increased levels of tyrosinated alpha-, beta(III)-, and beta(IV)-tubulin isotypes in paclitaxel-resistant MCF-7 breast cancer cells Biochem Biophys Res Commun 2002 293 598 601 10.1016/S0006-291X(02)00269-3 12054644

[B25] Ferlini C Raspaglio G Mozzetti S Cicchillitti L Filippetti F Gallo D Fattorusso C Campiani G Scambia G The seco-taxane IDN5390 is able to target class III beta-tubulin and to overcome paclitaxel resistance Cancer Res 2005 65 2397 2405 10.1158/0008-5472.CAN-04-3065 15781655

[B26] Gan PP Pasquier E Kavallaris M Class III beta-tubulin mediates sensitivity to chemotherapeutic drugs in non small cell lung cancer Cancer Res 2000 67 9356 9363 10.1158/0008-5472.CAN-07-0509 17909044

[B27] Ranganathan S Dexter DW Benetatos CA Chapman AE Tew KD Hudes GR Increase of beta(III)- and beta(IVa)-tubulin isotopes in human prostate carcinoma cells as a result of estramustine resistance Cancer Res 1996 56 2584 2589 8653701

[B28] Huzil JT Winter P Johnson L Weis AL Bakos T Banerjee B Luduena RF Damaraju S Tuszynski JA Modification of colchicine cytotoxicity and selectivity through the rational design of novel derivatives Chemical Biology and Drug Design 2010 in press 10.1111/j.1747-0285.2010.00970.x 20408852

[B29] Jaynes ET Information Theory and Statistical Mechanics Phys Rev 1957 106 620 630 10.1103/PhysRev.106.620

[B30] Shore JE Johnson RW Axiomatic Derivation of the Principle of Maximum Entropy and the Principle of Minimum Cross-Entropy Inf Theory 1980 IT-26 26 37 10.1109/TIT.1980.1056144

[B31] Shore JE Johnson RW Properties of Cross-Entropy Minimization IEEE Trans Inf Theory 1981 IT-27 472 482 10.1109/TIT.1981.1056373

[B32] Skilling J Erickson G, Smith CR The Axioms of Maximum Entropy, in Maximum Entropy and Bayesian Methods 1988 Dordrecht, Kluwer 173 187

[B33] Skilling J Skilling J, Dordrecht, Kluwer Classic Maximum Entropy Maximum Entropy and Bayesian Methods 1989 45 52

[B34] Skilling J Fougere PF Quantified Maximum Entropy Maximum Entropy and Bayesian Methods 1990 Dordrecht, Kluwer 341 350

[B35] Caticha A Erickson G, Zhai Y Relative Entropy and Inductive Inference Maximum Entropy and Bayesian Methods in Science and Engineering 2004 707 AIP Conf. Proc. (Melville, New York) 75 96

[B36] Mane JY Klobukowskij M Huzil T Tuszynski J Free Energy Calculations on the Binding of Colchicine and Its Derivatives with the *α*/*β*-Tubulin Isoforms J Chem Info Mod 2008 48 1824 1832 10.1021/ci800054n 18712858

[B37] Schaftenaar G Noordik JH Molden: a pre- and post-processing program for molecular and electronic structures J Comput-Aided Mol Design 2000 14 123 134 10.1023/A:1008193805436 10721501

[B38] Schmidt MW Baldridge KK Boatz JA Elbert ST Gordon MS Jensen JH Koseki S Matsunaga N Nguyen KA Su S Windus TL Dupuis M Montgomery JA General atomic and molecular electronic structure system J Comput Chem 1993 14 1347 1363 10.1002/jcc.540141112

[B39] Dewar M Zoebisch E Healy E Stewart J AM1: A new general purpose quantum mechanical molecular model J Am Chem Soc 1985 107 3902 3909 10.1021/ja00299a024

[B40] Dewar M Dieter K Evaluation of AM1 calculated proton affinities and deprotonation enthalpies J Am Chem Soc 1986 108 8075 8086 10.1021/ja00285a033

[B41] Field MJ Albe M Bret C Martin F Thomas A The DYNAMO library for molecular simulations using hybrid quantum mechanical and molecular mechanical potentials J Comput Chem 2000 21 1088 1100 10.1002/1096-987X(200009)21:12<1088::AID-JCC5>3.0.CO;2-8

[B42] Tembe B McCammon J Ligand-receptor interactions Comput Chem 1984 8 281 283 10.1016/0097-8485(84)85020-2

[B43] Motulsky H Christopoulos A Fitting Models to Biological Data using Linear and Nonlinear Regression 2004 New York NY: Oxford University Press

[B44] Tian G Haffner CD Linear relationships between the ligand binding energy and the activation energy of time-dependent inhibition of steroid 5α-reductase by Δ^1^-4-Azasteroids J Bio Chem 2001 276 21359 21364 10.1074/jbc.M100793200 11279132

[B45] Jaynes E Probability Theory: The Logic of Science 2003 Cambridge UK: Cambridge University Press

[B46] Khan IA Ludueña RF Different effects of vinblastine on the polymerization of isotypically purified tubulins from bovine brain Invest New Drugs 2003 21 3 13 10.1023/A:1022946305242 12795525

[B47] Yeh IT Ludueña RF The beta II isotype of tubulin is present in the cell nuclei of a variety of cancers Cell Motil Cytoskeleton 2004 57 96 106 10.1002/cm.10157 14691949

[B48] Burgoyne RD Cambray-Deakin MA Lewis SA Sarkar S Cowan NJ Differential distribution of beta-tubulin isotypes in cerebellum EMBO J 1988 7 2311 2319 2461292 10.1002/j.1460-2075.1988.tb03074.x PMC457095

[B49] Rowinsky EK Eisenhauer EA Chaudhry V Arbuck SG Donehower RC Clinical toxicities encountered with paclitaxel Semin Oncol 1993 20 1 15 8102012

[B50] Pratt WB Ruddon RW Ensminger WD Maybaum J The Anticancer Drugs 1994 2 NY. Oxford University Press p 191

[B51] Wolf S Barton D Kottschade L Grothey A Loprinzi C Chemotherapy-induced peripheral neuropathy: prevention and treatment strategies Eur J Cancer 2008 44 1507 1515 10.1016/j.ejca.2008.04.018 18571399

[B52] Katsetos CD Del Valle L Geddes JF Assimakopoulou M Legido A Boyd JC Balin B Parikh NA Maraziotis T de Chadarevian JP Varakis JN Matsas R Spano A Frankfurter A Herman MM Khalili K Aberrant localization of the neuronal class III beta-tubulin in astrocytomas. A marker for anaplastic potential Arch Pathol Lab Med 2001 125 613 624 11300931 10.5858/2001-125-0613-ALOTNC

[B53] Katsetos CD Del Valle L Geddes JF Aldape K Boyd JC Legido A Khalili K Perentes E Mörk SJ Localization of the neuronal class III beta-tubulin in oligodendrogliomas: comparison with Ki-67 proliferative index and 1p/19q status J Neuropathol Exp Neurol 2002 61 307 320 11939586 10.1093/jnen/61.4.307

[B54] Katsetos CD Legido A Perentes E Mörk SJ Class III beta-tubulin isotype: a key cytoskeletal protein at the crossroads of developmental neurobiology and tumor neuropathology J Child Neurol 2003 18 851 866 10.1177/088307380301801205 14736079

[B55] Dumontet C Isaac S Souquet PJ Bejui-Thivolet F Pacheco Y Peloux N Frankfurter A Ludueña RF Perol M Expression of class III beta tubulin in non-small cell lung cancer is correlated with resistance to taxane chemotherapy Elect J Oncol 2002 1 58 64 15749640

[B56] Banerjee A Roach MC Wall KA Lopata MA Cleveland DW Ludueña RF A monoclonal antibody against the type II isotype of beta-tubulin. Preparation of isotypically altered tubulin J Biol Chem 1988 263 3029 3034 3277964

[B57] Drewes E Ebneth A Mandelkow EM MAPs, MARKs and microtubule dynamics Trends Biochem Sci 1998 23 307 311 10.1016/S0968-0004(98)01245-6 9757832

[B58] Ferlini C Raspaglio G Cicchilitti L Mozzetti S Prislei S Bartolino S Scambia S Looking at drug resistance mechanisms for microtubule interacting drugs: does TUBB3 work? Curr Cancer Drug Targets 2007 7 704 712 10.2174/156800907783220453 18220531

[B59] Pepe A Sun L Zanardi I Wu X Ferlini C Fontana G Bombardelli E Ojima I Novel C-seco-taxoids possessing high potency against paclitaxel-resistant cancer cell lines overexpressing class II beta-tubulin Bioorg Med Chem Lett 2009 19 3300 3304 10.1016/j.bmcl.2009.04.070 19423340 PMC2700829

[B60] Dumontet C Jordan MA Lee FFY Ixabepilone: targeting betaIII-tubulin expression in taxane- resistant malignancies Mol Cancer Ther 2009 8 17 25 10.1158/1535-7163.MCT-08-0986 19139109

[B61] Cianfrocca M Application of epothilones in breast cancer therapy Curr Opin Oncol 2008 20 634 638 10.1097/CCO.0b013e32831270b0 18841044

[B62] Gupta D Mani S The efficacy and safety of ixabepilone monotherapy in the treatment of breast and gynecologic malignancies Expert Opinion on Drug Safety 2009 8 81 88 10.1517/14740330802655538 19236220

